# ZipV Is Required for Oxidative Stress Resistance and Pathogenicity in *Aspergillus fumigatus*

**DOI:** 10.3390/jof12050337

**Published:** 2026-05-05

**Authors:** Kinga Edina Varga, Zsigmond Benkő, Károly Antal, Brigitta Povazsanyecz, Katalin Forgács, István Pócsi, Tamás Emri

**Affiliations:** 1 Department of Molecular Biotechnology and Microbiology, Institute of Biotechnology, Faculty of Science and Technology, University of Debrecen, H-4032 Debrecen, Hungary; kinga_varga@yahoo.com (K.E.V.); benko.zsigmond@science.unideb.hu (Z.B.); brigipova@gmail.com (B.P.); forgacskatalin22@gmail.com (K.F.); 2 Doctoral School of Nutrition and Food Sciences, University of Debrecen, H-4032 Debrecen, Hungary; 3 Department of Zoology, Eszterházy Károly Catholic University, H-3300 Eger, Hungary; antalk2@gmail.com; 4 HUN-REN–UD Fungal Stress Biology Research Group, H-4032 Debrecen, Hungary

**Keywords:** *Aspergillus fumigatus*, bZIP transcription factors, *Galleria mellonella*, oxidative stress response, conidial development, virulence, ZipV

## Abstract

The functions of the putative bZIP (basic leucine zipper) transcription factors ZipV (Afu3g032301) and ZipZ (Afu2g14350) were investigated using wild-type, gene-deficient, and complemented strains of *Aspergillus fumigatus*. Deletion of *zipV* increased oxidative stress sensitivity and reduced in vivo virulence in a *Galleria mellonella* model, whereas complementation restored the wild-type phenotype. In contrast, deletion of *zipZ* resulted in no detectable phenotypic changes, even though transcription of both genes was modulated by oxidative stress. Phenotypic characterization of conidia, transcriptomic analyses of growing cultures and reverse-transcription quantitative real-time PCR of conidia-producing cultures suggested that ZipV regulates the development of certain conidial traits. The conidia of the ∆*zipV* mutant showed reduced heat stress tolerance, decreased catalase activity and delayed germination in comparison to the wild-type or the complemented strain. In the ∆*zipV* mutant, the transcription of *catA* encoding conidial catalase was impaired. This defect explains the reduced catalase activity and the oxidative stress sensitivity of the mutant and may contribute to its reduced virulence. The increased transcriptional activity of the alternative oxidase gene *aoxA* observed in the absence of ZipV suggests a compensatory response aimed at mitigating oxidative stress.

## 1. Introduction

*Aspergillus fumigatus* is a widespread soil saprotroph that can cause severe invasive disease in immunocompromised patients [1]. In recent years, the global risk of invasive aspergillosis has increased due to COVID-19–associated pulmonary aspergillosis and the expanding use of novel immunomodulatory and anticancer drugs [1]. Compounding this trend is the rising resistance to triazoles, which remain the first-line therapy for invasive aspergillosis [2]. Because of these factors—together with high mortality rates and diagnostic limitations—*A*. *fumigatus* has been added to the WHO’s list of priority fungal pathogens [3].

Several characteristics of *A. fumigatus* that may contribute to its survival in the host have been investigated [1,4,5]. Among these, oxidative stress tolerance remains one of the most debated. Phagocytosis (and frustrated phagocytosis) mediated by macrophages and neutrophils, as well as uptake by airway epithelial cells, are key components of the innate immune response to *A. fumigatus* infection [6]. Because the killing activity of these cells partly depends on the formation of reactive oxygen species (ROS), oxidative stress tolerance is considered to be important for fungal pathogens. Data indicate that the iron-limited environment of the human body compromises the oxidative stress tolerance of fungi [7,8], underscoring the requirement for potent antioxidant defenses for survival in the host. However, studies conducted on *A. fumigatus* mutants with altered ROS elimination have produced inconsistent results: Deficiency in OxrA (oxidation resistance 1 protein), Aspf3 (peroxiredoxin), or Pes1 (non-ribosomal peptide synthase) increased oxidative-stress sensitivity and resulted in hypovirulence, whereas deletion of three fatty acid oxygenase genes (*ppoA*, *ppoB*, and *ppoC*) enhanced oxidative-stress tolerance and was associated with hypervirulence [9,10,11,12]. In contrast, neither the Δ*sod1*Δ*sod2*Δ*sod3* triple-deletion superoxide dismutase mutant, the Δ*catA* catalase mutant, nor mutants deficient in regulatory elements of the oxidative stress response—such as the Sho1 sensor; MpkA or PkcA protein kinases; or the Skn7 and Yap1 transcription factors—showed reduced virulence in in vivo models, despite displaying reduced oxidative stress tolerance [13,14,15,16,17,18,19]. Even the Δ*cat1*Δ*cat2* double-deletion catalase mutant exhibited only slightly attenuated virulence, together with moderately increased oxidative stress sensitivity [13].

These seemingly contradictory outcomes suggest that virulence is not tightly coupled to oxidative-stress tolerance. Several factors may explain this: (1) The oxidative stress defense system of *A. fumigatus*—while not stronger than that of most medically less relevant *Aspergillus* species [20,21]—is highly redundant [22]. (2) Even subtle alterations in stress tolerance are readily detectable in vitro (and although such changes may indeed affect in vivo virulence), the pathogenicity assays may lack the sensitivity to resolve these minor shifts in virulence. (3) Strains with reduced oxidative stress tolerance can still survive in infected rodents because most animals used in aspergillosis models are leukopenic [23]. (4) Oxidative stress tolerance factors identified in vitro may not play major roles in vivo [8].

bZIP (basic leucine zipper) transcription factors constitute one of the oldest transcription factor families, originating from a single ancestral eukaryotic gene. Members of this family function either as homodimers or as heterodimers formed with other bZIP-type transcription factors. In fungi, bZIP proteins play central roles in the regulation of primary metabolism, cellular differentiation, stress responses, virulence, and secondary metabolite biosynthesis [24]. In *A. fumigatus*, well-characterized bZIP transcription factors include HapX, which controls iron acquisition and adaptation to iron-limited conditions [25]; Yap1, which is the key transcription factor of oxidative stress response [15]; AtfA and AtfB, which are required for oxidative, hyperosmotic and cell wall stress tolerance and are also involved in the regulation of carbon metabolism [26,27,28]; and MeaB, which regulates nitrogen metabolism and contributes to proper nitrosative and cell wall stress responses [29,30]. Not surprisingly, deletion of *hapX*, *atfA*, and *atfB*, but not *yap1* genes, was accompanied by decreased virulence in various in vivo infection models, while in the case of *meaB*, this property was strain specific [15,25,28,29,30].

In a previous study, we used a transcriptome-based approach to examine how the oxidative stress response of *A. fumigatus* depends on culture conditions, specifically glucose and iron availability [8]. We found only limited overlap among antioxidant enzyme genes upregulated by H_2_O_2_ across different conditions. These findings support the view that proteins identified as important for oxidative stress resistance in vitro are not necessarily essential in vivo, and therefore the loss of these proteins does not necessarily affect virulence. The transcriptomic data drew our attention to two genes encoding putative bZIP transcriptional factors: Afu3g03230 (*zipV)* and Afu2g14350 (*zipZ*). H_2_O_2_ selectively upregulated *zipV* under glucose-free and *zipZ* under iron-limited conditions [8], suggesting that their involvement in oxidative stress regulation is more context-specific than that of Yap1, a key oxidative stress response transcription factor in *A. fumigatus* [15]. Here, we examined *zipV* and *zipZ* gene deletion mutants and found that, unlike ZipZ, ZipV enhances protection against oxidative stress induced by either H_2_O_2_ or *tert*-butyl hydroperoxide (tBOOH) and also increases in vivo virulence in the *G. mellonella* model.

## 2. Materials and Methods

### 2.1. Strains

*A. fumigatus* Af293 (wild-type strain), VkzipV1 (the *zipV* gene-deletion mutant of Af293), VkzipV2 and VkzipV22 (the *zipV*-complemented strains of VkzipV1), and VkzipZ1 (the *zipZ* gene-deletion mutant of Af293) were studied. Strains were maintained on Barratt’s minimal agar plates [31] at 37 °C. Conidia, freshly isolated from 6-day-old cultures with phosphate-buffered saline (PBS) solution also containing 0.01 *v*/*v*% Tween 80, were used for all experiments.

### 2.2. Construction of the zipV and zipZ Gene Deletion and the zipV Complementation Strains

The *zipV* and *zipZ* knockout strains (VkzipV1 and VkzipZ1) were constructed by replacing the respective genes (Afu3g03230 and Afu2g14350) of the *A. fumigatus* Af293 strain with a hygromycin resistance cassette. For site-specific homologous recombination, the *hph* gene from the pAN7.1 plasmid (AddGene, Watertown, MA, USA) was flanked in both cases by two, approximately 1.5 kb fragments homologous to the upstream and downstream sequences of the target gene. For the construction of the complemented *zipV* strains (VkzipV2 and VkzipV22), the original *zipV* gene, including its native promoter and terminator—from the Af293 strain—was introduced into the genome of the VkzipV1 gene deletion mutant. The *zipV* gene was fused to the *ble* gene from the pFC333 plasmid (AddGene, Watertown, MA, USA), which carries the *A. nidulans trpC* promoter. In the case of all constructs, the respective fragments were assembled into the pYTK001 plasmid (AddGene, Watertown, MA, USA) using the NEBuilder HiFi DNA Assembly Kit (New England Biolabs, Ipswich, MA, USA). Electroporation-based transformation of conidia was performed as described previously [30]. PCR was used to verify the successful integration of the deletion cassette and the presence of the complementation cassette, and reverse-transcription quantitative real-time PCR (RT-qPCR) was used to check the expected absence or presence of the gene expression. Primer pairs used for constructing the gene-deletion and complementation strains as well as the results of the PCR- and RT-qPCR-based validation experiments are listed in Tables S1 and S2 and Figure S1.

### 2.3. Characterizing Stress Susceptibility

Stress susceptibility of the strains was tested on Barratt’s minimal agar plates. Cultures were point-inoculated (5 µL of 1 × 10^5^ conidia/mL) and incubated at 37 °C for 5 days. Stress susceptibility was assessed based on stressor-induced reductions in colony diameter. The following stressors were studied: menadione sodium bisulfite (MSB; 6 µM), H_2_O_2_ (0.5 mM), *tert*-butyl-hydroperoxide (tBOOH; 0.8 mM), sorbitol (1 M), NaCl (0.5 M), Congo red (15 mM), deferiprone (DFP; 1 mM), ZnSO_4_ (8 mM), FeCl_3_ (6.5 mM), CuCl_2_ (0.5 mM), and CdCl_2_ (2 mM). For DFP treatment (iron chelation), iron was omitted from the medium. All experiments were performed with three biological replicates, and differences between group means were assessed using a two-sided Student’s *t*-test.

### 2.4. Characterization of Conidia

The size of conidia was determined by light microscopy. A total of 150 conidia isolated from three independent plates were measured. Germination of conidia (0.5 × 10^6^ conidia/mL) was followed for 10 h in Barratt’s minimal broth at 37 °C, and the ratio of the germinated conidia was determined by light microscopy using three biological replicates. The heat tolerance of conidia was evaluated at 55 °C for 15 min following a previously described method [26] by comparing the number of colony-forming units obtained from treated and untreated cultures in three biological replicates. For the measurement of catalase activity in conidia, the conidia were disrupted using glass beads. Catalase activity was determined from the cell-free extract prepared by centrifugation (10 min, 10,000× *g*, 4 °C). The rate of H_2_O_2_ consumption was monitored spectrophotometrically at 240 nm as described previously [32]. Catalase activity was expressed as mkat/kg protein. Protein concentration of the samples was determined using the Bradford reagent.

All conidium-specific traits were assessed using three biological replicates, and differences between group means were evaluated using a two-sided Student’s *t*-test.

### 2.5. Galleria mellonella Infection Model

*G. mellonella* larvae were obtained from Bugs World (Budapest, Hungary). Actively moving, non-melanized larvae (weighing 0.2–0.4 g) were administered either with 10 µL PBS (phosphate-buffered saline) containing 5 × 10^5^ conidia or 10 µL PBS as reference via the last right proleg using an insulin syringe (G32). Larvae were kept in small (55 mm-diameter) Petri dishes (5 larvae per dish) in the dark at 37 °C, and their survival was monitored daily for one week. Both the infected and reference groups contained 20 randomly selected larvae, and the experiment was carried out in duplicate. Data were evaluated with the log-rank test using the “survdiff” function of the survival package in R (https://CRAN.R-project.org/package=survival (accessed on 3 March 2025)).

### 2.6. High Throughput RNA Sequencing

The transcriptomes of the VkzipV1 (∆*zipV*) and Af293 (reference) strains were analyzed in submerged cultures using three biological replicates. Barratt’s minimal liquid medium supplemented with 5 g/L yeast extract was prepared, after which 100 mL aliquots were dispensed into 500 mL Erlenmeyer flasks, inoculated with 5 × 10^7^ conidia, and incubated at 37 °C and 220 rpm (approximately 3.7 Hz) for 17 h, to obtain cultures in the exponential growth phase. Mycelia from 50 mL of these cultures were then transferred to 100 mL of minimal liquid media containing 4 g/L casein peptone, 1.52 g/L KH_2_PO_4_, 0.52 g/L MgSO_4_ 4 H_2_O, 0.52 g/L KCl, and iron-free Barratt’s trace elements solution (pH 6.5). Cultures were incubated at 37 °C and 220 rpm; after 8 h, a subset was treated with 90 mM H_2_O_2_, followed by an additional 1 h of incubation. Total RNA was isolated from lyophilized mycelia using the acid guanidinium thiocyanate-phenol-chloroform extraction method [33]. RNA integrity was assessed with the Eukaryotic Total RNA Nano kit on an Agilent Bioanalyzer (Agilent Technologies, Inc., Santa Clara, CA, United States). Illumina RNA sequencing was performed at the Genomic Medicine and Bioinformatic Core Facility (University of Debrecen, Debrecen, Hungary). Libraries were prepared with the TruSeq RNA Sample Preparation Kit (Illumina, Inc., San Diego, CA, United States). All library pools (two strains × two conditions × three biological replicates = 12 pools) were sequenced in a single flow cell lane. Reads (14.6–21.9 million reads/sample) were aligned to the *A. fumigatus* Af293 genome using hisat2 (version 2.2.1; [34]) with genome and feature data from FungiDB (release 68: https://fungidb.org/common/downloads/release-68/AfumigatusAf293/fasta/data/FungiDB-68_AfumigatusAf293_Genome.fasta as genome data (accessed on 3 March 2025) and https://fungidb.org/common/downloads/release-68/AfumigatusAf293/gff/data/FungiDB-68_AfumigatusAf293.gff (accessed on 3 March 2025)). Alignment efficiency ranged from 94.5% to 95.5%. Read counts and RPKM (reads per kilobase per million) values were calculated with featureCounts 2.0.6 [35] and the “rpkm” function from the package edgeR, while differentially expressed genes (DEGs; adjusted *p* < 0.05) were identified with DESeq2 1.40.2 [36].

### 2.7. Evaluation of Transcriptome Data

Principal component analysis (PCA) was used to visualize similarities among transcriptomes. PCA was performed using the “prcomp” function of the R project (r-project.org) on rlog-transformed count data generated by DESeq2 (version 1.40.2).

Gene set enrichment analyses were performed using ShinyGO 0.77 (http://bioinformatics.sdstate.edu/go77/ (accessed on 1 December 2025)) on DEGs with |log_2_FC| > 1, applying the default settings for KEGG pathways and Gene Ontology (GO) terms. Enrichments with FDR-corrected *p* < 0.05 were considered significant.

Enrichment of selected stress-related gene groups in the up- or downregulated gene sets was also tested with Fisher’s exact test (“fisher.test” in R; r-project.org/). The following gene groups were examined: “Antioxidative enzyme”, and “Glutathione metabolism” genes [37]; “Respiration” genes (KEGG pathway database; https://www.kegg.jp/pathway/afm00190 (accessed on 1 March 2025)); “Reductive iron assimilation (RIA)” genes [38]; and “Siderophore cluster” genes [39].

### 2.8. Comparing Relative Transcriptional Activity of Genes in Growing and Conidia-Producing Submerged Cultures by RT-qPCR Assay

The relative transcriptions of the *zipV* transcription factor gene as well as *catA* (encoding conidial catalase; [13]) and *rodA* (encoding conidial hydrophobin; [40]) were compared in growing and conidia-forming submerged cultures of the VkzipV1 (∆*zipV*), VkzipV2 (*zipV*-complemented strain), and Af293 (reference) strains, each with three biological replicates.

To obtain conidia-producing fungal cultures under submerged conditions, Barratt’s minimal liquid medium [31] was inoculated with 5 × 10^5^ conidia/mL and incubated at 37 °C with shaking at 220 rpm for 17 h before sampling.

To obtain growing but not conidia-producing cultures, Barratt’s minimal liquid medium supplemented with 5 g/L yeast extract was inoculated with 5 × 10^5^ conidia/mL and incubated at 37 °C and 220 rpm for 17 h; mycelia were harvested, transferred to fresh medium, and incubated for an additional 4 h.

Total RNA was isolated from lyophilized mycelia [33]. RT-qPCR reactions were performed with the Luna Universal One-Step RT-qPCR Kit (New England Biolabs, Ipswich, MA, USA). Relative transcription was quantified using the ΔCP value: ΔCP = CP_reference_ – CP_target_, where CP is the crossing point of the reaction. The *tef1* (Afu1g06390) gene encoding the translation elongation factor α-subunit was used as a reference. Primer pairs are listed in Table S1. Significant differences between the ∆CP values for growing vs. conidia-producing cultures of the same strain were assessed using a two-sided Student’s *t*-test.

## 3. Results and Discussion

### 3.1. ZipV Contributed to Oxidative Stress Tolerance and Virulence in A. fumigatus

**Figure 1:**
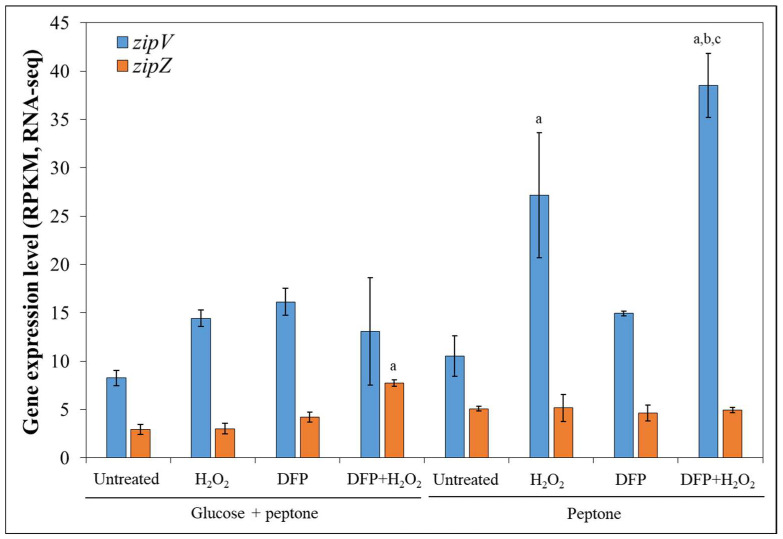
Transcriptional behavior of *zipV* (blue) and *zipZ* (brown) in submerged *A. fumigatus* Af293 cultures. Exponential-phase mycelia were cultivated in media containing glucose-peptone or peptone alone as the carbon source in the presence or absence of 1 mM deferiprone (DFP). After 8 h of incubation, some cultures were treated with 75 mM H_2_O_2_. Gene expression levels were quantified by high-throughput RNA sequencing and were visualized as RPKM values calculated by the “rpkm” function from the package edgeR (mean ± S.D.; *n* = 3). Transcriptional differences caused by H_2_O_2_ treatment, DFP pretreatment, or glucose omission were assessed, and differentially expressed genes (DEGs) were determined with DESeq2. For further details see Emri et al. (2024) [8]. The figure was prepared using Microsoft Excel. ^a,b,c^—The gene was classified as a DEG with a transcriptional difference greater than twofold (|log_2_FC| > 1) when compared to the corresponding “Untreated” culture (^a^), “DFP”-treated culture (^b^), or glucose-containing culture (^c^).

The function of two *A. fumigatus* genes (Afu3g03230; *zipV* and Afu2g14350; *zipZ*), which putatively encode transcription factors, was examined. Both *zipV* and *zipZ* are part of the *A. fumigatus* core genome [41] and encode proteins containing a bZIP domain (FungiDB; https://fungidb.org/fungidb/app/ (accessed on 1 March 2025)). These genes were selected based on their context-dependent transcriptional behavior observed in a previous transcriptomics study [8]. The *zipV* gene was upregulated by H_2_O_2_ treatment in submerged *A. fumigatus* Af293 cultures when peptone served as the carbon source—whereas this response was absent in glucose-containing media—regardless of DFP pretreatment (Figure 1). In contrast, *zipZ* was induced by H_2_O_2_ only under glucose-based growth conditions and only following DFP pretreatment (Figure 1). To better understand their respective contributions to *A. fumigatus* stress responses, gene-deletion (and complemented) strains were generated and characterized. MSB reduced the colony diameter of the ∆*zipV* gene deletion mutant culture slightly but significantly more than that of either the reference strain or the complemented mutant (Table 1).

**Figure 2:**
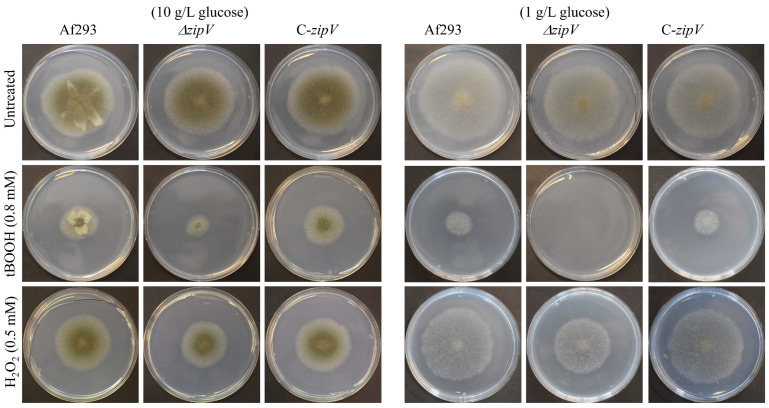
H_2_O_2_ and tBOOH stress tolerance of *A. fumigatus* ∆*zipV* mutant. Representative images (taken after 5 days incubation) of the reference strain (Af293), the *ΔzipV* gene deletion mutant (VkzipV1), and the complemented strain (*C-zipV*; VkzipV2) from three biological replicates are shown. Petri dish diameter: 85 mm. The figure was prepared using Microsoft PowerPoint.

For oxidative stresses elicited by H_2_O_2_ or tBOOH, the differences were much more pronounced, especially on media containing 1 g/L glucose (instead of the usual 10 g/L) (Table 1, Figure 2). For all other stressors tested, no significant differences were observed between the gene deletion mutant and the reference strain (Table S3). With respect to the *zipZ* gene, no significant differences were detected between the *zipZ* deletion mutant and the reference strain (Table S4).

**Figure 3:**
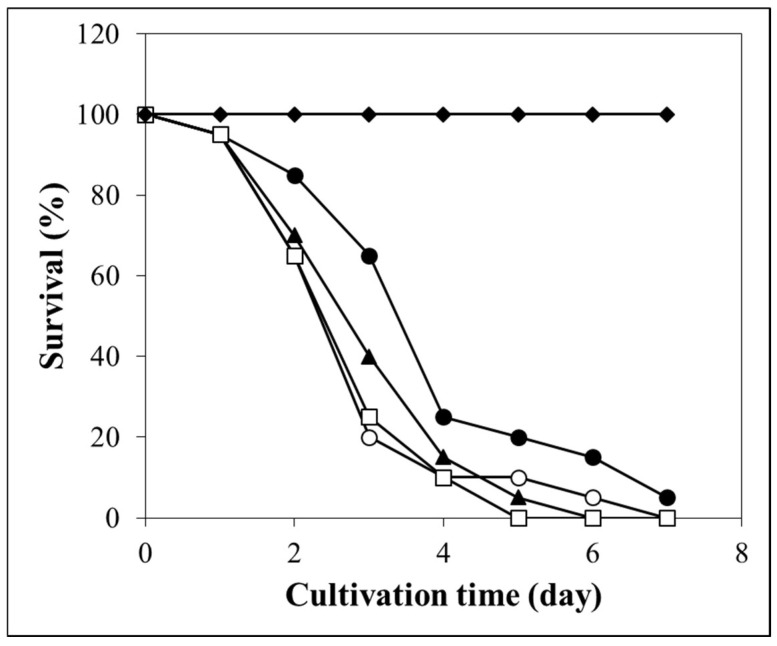
In vivo virulence of *A. fumigatus* conidia in the *G. mellonella* model. Data with the ∆*zipV* (VkzipV1; closed circle ●), C*-zipV* (VkzipV2; open circle ○), ∆*zipZ* (VkzipZ1; closed triangle ▲), and the Af293 reference strain (open square □) are presented. Larvae injected with 10 µL PBS were used as a negative control (closed diamond ♦). The experiment was carried out with three biological replicates; one representative data set is shown. The log-rank test found a significant difference between ∆*zipV* (●) and both Af293 (□) (*p* = 0.01) and C-*zipV* (○) (*p* = 0.02). The figure was prepared using Microsoft Excel.

The increased oxidative stress sensitivity of the ∆*zipV* mutant was accompanied by attenuated virulence in the *G. mellonella* infection model (Figure 3). In contrast, deletion of the *zipZ* gene had no significant effect on virulence (Figure 3).

### 3.2. Transcriptomic Analysis of the ΔzipV Mutant Reveals Limited Differential Expression Despite aoxA Upregulation

**Figure 4:**
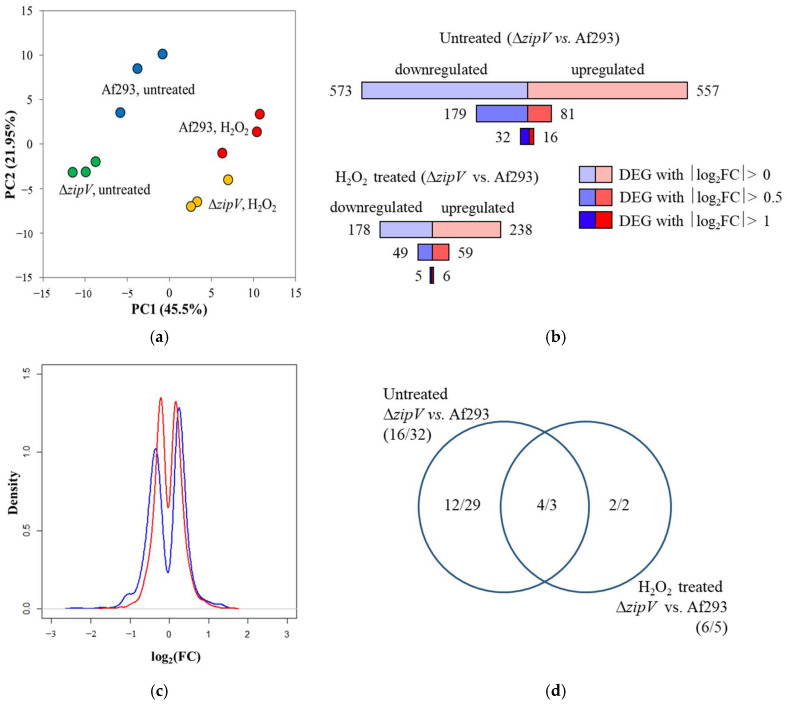
Characterization of differences between the transcriptomes of the ∆*zipV* and Af293 strains under untreated and H_2_O_2_-treated conditions. (**a**) Principal component (PC) analysis of the rlog values generated during the evaluation of RNAseq data. (**b**) Number of DEGs in the |log_2_FC| > 1, |log_2_FC| > 0.5 and |log_2_FC| > 0 gene sets under untreated and H_2_O_2_-treated conditions. (**c**) Density distribution of log_2_FC values for genes identified as DEGs in at least one of the two cultures (blue: untreated cultures, red: H_2_O_2_ treated). (**d**) Overlap between the upregulated and downregulated DEGs with |log_2_FC| > 1. Panel a was prepared using Microsoft Excel; panels b and d were prepared using Microsoft PowerPoint; and panel c was generated using the plotting functions of the R statistical environment.

The basis of the oxidative-stress-sensitive phenotype of the ∆*zipV* mutant was further investigated using transcriptome data. In these experiments, the exponentially growing-phase mycelia of the Af293 reference strain and the ∆*zipV* gene deletion mutant were cultivated in iron-limited minimal media containing casein peptone as the carbon and energy source, then either treated with H_2_O_2_ or left untreated (Figure 4a). Comparison of the transcriptomes of the two strains showed that the lack of ZipV caused only small differences under untreated conditions, and the H_2_O_2_ exposure further reduced these differences (Figure 4a–d). These findings suggest that ZipV plays only a modest role in regulating redox homeostasis under the tested conditions and/or its function can be compensated for by other regulatory factors.

**Figure 5:**
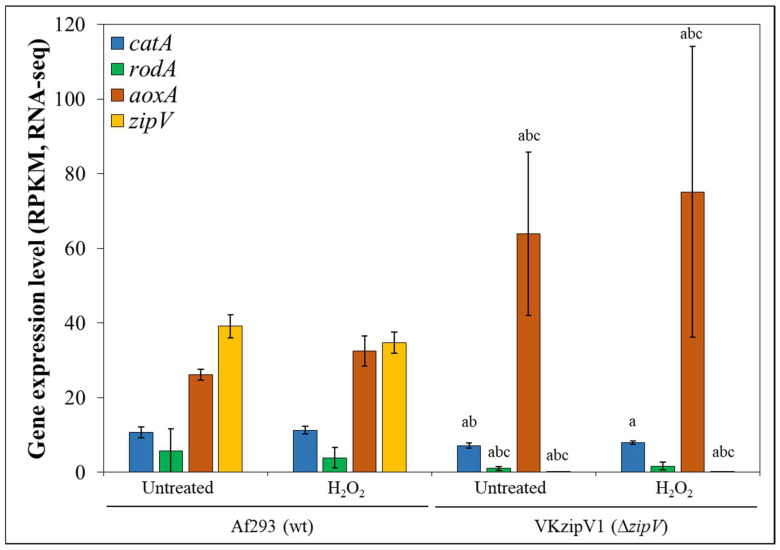
Transcriptional behavior of *catA* (blue), *rodA* (green), *aoxA* (brown), and *zipV* (orange) in submerged cultures of *A. fumigatus* Af293 (wt) and VKzipV1 (∆*zipV*). Exponential-phase mycelia were cultivated in media containing peptone as a carbon source. After 8 h of incubation, some of the cultures were treated with 90 mM H_2_O_2_. Gene expression levels were quantified by high-throughput RNA sequencing and visualized using RPKM values calculated by the “rpkm” function from the package edgeR (mean ± S.D.; *n* = 3). Differentially expressed genes (DEGs) between the two strains and between the H_2_O_2_-treated and untreated cultures were identified using DESeq2. The figure was prepared using Microsoft Excel. ^a,b,c^—When the transcriptional activity of a marked gene was compared between the two strains, the gene was grouped according to DEG status: (^a^) DEG, (^b^) DEG with |log_2_FC| > 0.5, or (^c^) |log_2_FC| > 1.

Gene set enrichment analysis of DEGs with |log_2_FC| > 1 did not yield additional insights, likely due to the limited number of up- and downregulated genes (Figure 4d, Table S5). We then examined the transcriptional profiles of genes belonging to selected functional groups (“Antioxidative enzyme”, “GSH metabolism”, “Respiration”, “RIA”, and “Siderophore cluster”; Table S5), which are commonly responsive to oxidative stress. Furthermore, we analyzed the known or putative functions of DEGs exhibiting at least twofold transcriptional differences (|log_2_FC| > 1) between the two strains (Table S5). Since transcriptional differences between the reference strain and the mutant were minor when hyphae were examined in submerged cultures (Figure 4), conidia-specific genes were of particular interest, as oxidative stress sensitivity of the Δ*zipV* mutant was observed in surface cultures inoculated with conidia (Figure 2). Finally, based on these analyses, we selected three genes (*catA*, *rodA*, and *aoxA*) as candidates for further investigation (Figure 5).

CatA encodes a conidium-specific catalase; its deletion increases conidial H_2_O_2_ sensitivity but does not alter resistance to macrophage killing or in vivo virulence [13]. The *rodA* gene encodes a hydrophobin that masks Dectin-1 and Dectin-2 recognition of conidia, thereby enhancing fungal survival in vivo [42]. AoxA encodes the alternative oxidase of the *A. fumigatus* respiratory chain; loss of this protein increases oxidative stress sensitivity, elevates ROS production, and reduces protection from macrophage killing, although virulence in murine models remains unaffected [43,44].

Based on these data, we propose the following: (i) The reduced oxidative stress tolerance (Table 1, Figure 2) and decreased in vivo virulence (Figure 3) of Δ*zipV* conidia most likely result from impaired transcription of genes that determine conidial traits. (ii) The modest transcriptomic differences between wild-type and Δ*zipV* strains (in the exponential growth phase) (Figure 4) suggest that ZipV plays a less prominent role here than during conidiogenesis. (iii) Upregulation of *aoxA* (Table S5, Figure 5) likely provides partial compensation for the absence of ZipV (in the exponential growth phase), helping to explain the mild phenotype.

### 3.3. ZipV Affects the Properties of Conidia-Forming Cultures

**Figure 6:**
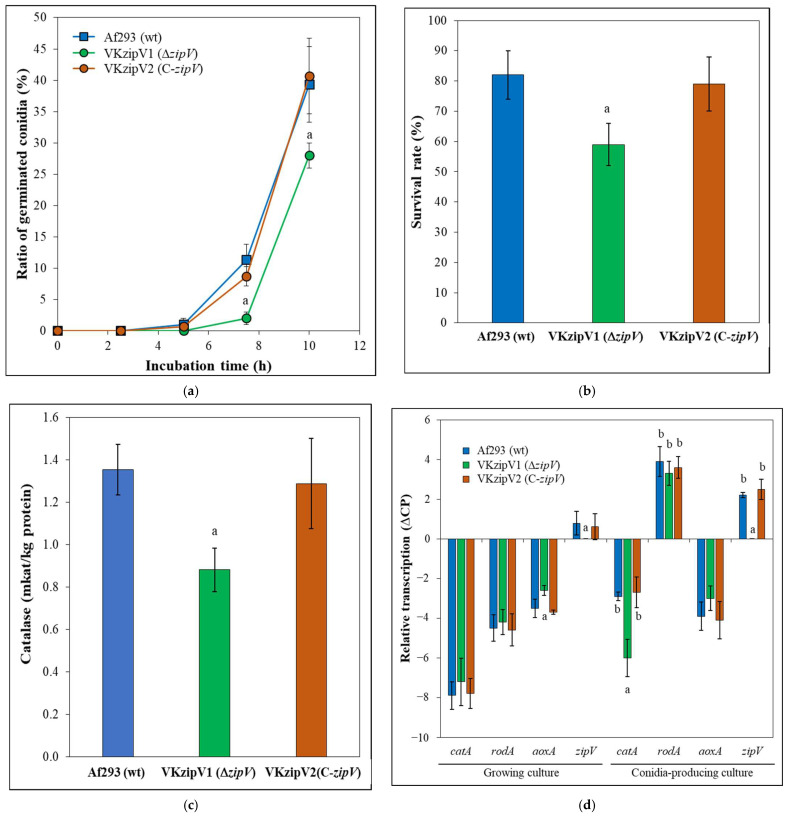
Comparison of some conidia-specific traits of the *A. fumigatus* Af293, ∆*zipV*, and C-*zipV* strains. Part (**a**): Germination of conidia in minimal nitrate broth. Part (**b**): Heat stress (15 min, 55 °C) tolerance of conidia. Part (**c**): Catalase activity of conidia. Part (**d**): Relative transcription levels of selected genes in growing and conidia-producing submerged cultures. Minimal nitrate broth was inoculated with conidia and incubated at 37 °C and 220 rpm for 17 h to obtain “conidia-producing cultures”. Exponentially growing, non-sporulating mycelia from rich liquid medium were transferred into minimal nitrate broth and incubated at 37 °C and 220 rpm for 4 h to obtain cultures that did not produce conidia (“growing culture”). Mean ± S.D. from three biological replicates are shown. The figure was prepared using Microsoft Excel. ^a^—Significant difference when compared to Af293 (under the same conditions) (Student’s *t*-test; *p* < 0.05). ^b^—Significant differences between the ∆CP values for growing vs. conidia-producing cultures of the same strain (Student’s *t*-test; *p* < 0.05).

To evaluate the role of ZipV in conidiogenesis, we characterized conidia from the VKzipV1, VKzipV2, and Af293 strains. Loss of ZipV did not affect conidial size (Figure S2); however, it resulted in delayed germination, increased conidial heat sensitivity, and reduced catalase activity compared with the complemented and wild-type strains (Figure 6a–c). By contrast, deletion of *atfA*, which encodes a transcription factor essential for conidial stress tolerance, led to more severe phenotypic effects [26]. In addition to their increased oxidative stress sensitivity, conidia of the Δ*atfA* strain failed to survive exposure to 55 °C for 15 min, exhibited a pronounced delay in germination, and showed increased size heterogeneity, including the formation of unusually large conidia in addition to normal-sized ones [26].

We further compared the relative transcript levels of *catA*, *rodA*, *aoxA*, and *zipV* in submerged cultures that either produced conidia or were maintained under non-conidiating conditions (Figure 6d). As expected, conidia production was associated with elevated *catA* and *rodA* transcription in both the wild-type and the complemented strain. Notably, *zipV* also showed increased transcription in conidia-producing cultures relative to the corresponding non-conidiating cultures of the wild-type and the complemented strain, supporting a role for ZipV in conidiogenesis. Loss of ZipV disturbed the upregulation of *catA* during conidia formation (in line with observed reduced conidial catalase activity; Figure 6c), further supporting a role for ZipV in the development of conidial properties. In contrast, *zipV* deletion did not affect the transcription of *rodA* (Figure 6d).

In previous work [13], the absence of CatA increased sensitivity to oxidative stress but did not attenuate virulence; however, conidial catalase is thought to play an important role in protecting conidia from the oxidative burst during phagocytosis. Although innate immune systems are conserved between mammals and insects, including functional and structural similarities between *G. mellonella* hemocytes and mammalian phagocytes, differences between these systems [45] may explain why the Δ*zipV* mutant—despite exhibiting increased sensitivity to oxidative stress, reduced *catA* expression, and lower catalase activity (Figure 2 and Figure 6)—showed decreased virulence in the *G. mellonella* in vivo model (Figure 3), whereas the Δ*catA* mutant does not display reduced virulence in macrophage assays or in a rat infection model [13]. Alternatively, immunosuppression in the rodent model may reduce selective pressure against oxidative stress sensitivity [23], enabling the Δ*catA* mutant to remain pathogenic, or the reduced virulence of the Δ*zipV* mutant may be linked to other, as yet unidentified, consequences of the loss of this bZIP transcription factor. Notably, the Δ*atfA* mutant, which also regulates *catA* expression, exhibited attenuated virulence in both the *G. mellonella* and neutropenic murine models, accompanied by decreased tolerance to osmotic and cell wall stress, in addition to reduced oxidative stress tolerance [28].

In addition, deletion of *zipV* increased the transcriptional activity of *aoxA*—consistent with the RNAseq data (Table S5, Figure 5)—although this increase was not significant in conidia-producing cultures. AoxA contributes to oxidative stress protection by decreasing ROS generation associated with respiration in several *Aspergillus* species, including *A. fumigatus* [44], *A. niger* [46], and *A. terreus* [47]. Although deletion of the *aoxA* (alternative oxidase) gene in *A. nidulans* did not alter the oxidative stress sensitivity of the fungus, it increased the ROS formation in glucose-supplemented cultures and was accompanied by elevated catalase and superoxide dismutase activities [48]. Previous studies have shown that *aoxA* is upregulated in CycA-deficient *A. fumigatus* mutants, where increased *aoxA* activity is accompanied by elevated resistance against oxidative stress and macrophage-mediated killing in vitro, yet does not prevent attenuation of in vivo virulence [44]. These findings, in line with our results, suggest that flexible regulation of *aoxA* enables cells to partially mitigate vulnerabilities caused by gene deletion. However, in conidia, where respiration operates at a very basal level, elevated *aoxA* transcription is unlikely to provide substantial oxidative stress protection. This may account for the increased oxidative stress sensitivity observed in surface cultures inoculated with conidia (Table 1), as well as the reduced virulence in the *G. mellonella* model (Figure 3), where infections were initiated with conidia.

Nevertheless, further characterization of the Δ*zipV* mutant is required, including the assessment of additional stress tolerance traits, such as antifungal susceptibility. Given the limitations of the *G. mellonella* model in extrapolating results to humans, evaluating the in vivo virulence of the Δ*zipV* mutant in alternative models, such as murine models, as well as assessing the susceptibility of conidia to human macrophage-mediated killing, could further elucidate the contribution of ZipV to virulence in humans.

## 4. Conclusions

Our results indicate that the ZipV transcription factor of *A. fumigatus* primarily influences conidial traits. ZipV may function similarly to AtfA in regulating conidial stress tolerance; however, ZipV likely controls a smaller subset of target genes, or its loss may be more readily compensated for by other transcription factors than the loss of AtfA. In the absence of ZipV, the conidial catalase gene *catA* was not properly upregulated during conidiogenesis, resulting in reduced conidial catalase activity, which explains the increased oxidative-stress sensitivity observed in cultures inoculated with conidia. Since the impaired transcription of *catA* alone cannot fully account for the reduced in vivo virulence of the Δ*zipV* strain observed in the *G. mellonella* model, it is reasonable to assume that ZipV may also influence the expression of additional, as yet unidentified genes, either directly or indirectly. Our findings also underscore that the functional loss of a single antioxidative gene can be masked (fully or partially) by compensatory upregulation of alternative antioxidative pathways—*aoxA* in this case. The extent of such compensation may depend on the culturing conditions. Consequently, mutants that appear phenotypically unremarkable under in vitro conditions may exhibit pronounced defects in vivo that ultimately impair virulence or, alternatively, may show the opposite trend.

## Figures and Tables

**Table 1:** Characterization of the oxidative stress tolerance of *A. fumigatus ∆zipV* strain.

		Colony Diameter (cm) ^a^			*p*-Value ^b^	
		Af293	∆*zipV*	*C-zipV*	∆*zipV*	*C-zipV*
10 g/L glucose	Untreated	5.8 ± 0.17	5.8 ± 0.06	5.7 ± 0.10	0.768	0.435
	MSB (6 µM)	4.4 ± 0.15	3.9 ± 0.06	4.3 ± 0.10	0.004	0.275
	tBOOH (0.8 µM)	3.2 ± 0.06	1.9 ± 0.10	3.3 ± 0.06	4.5 × 10^−5^	0.101
	H_2_O_2_ (0.5 mM)	4.7 ± 0.20	3.7 ± 0.35	4.7 ± 0.06	0.012	0.795
1 g/L glucose ^c^	Untreated	6.6 ± 0.15	6.3 ± 0.15	6.2 ± 0.25	0.074	0.078
	MSB (6 µM)	4.7 ± 0.15	4.2 ± 0.06	4.6 ± 0.06	0.004	0.152
	tBOOH (0.8 µM)	2.2 ± 0.15	0.0 ± 0.0	2.1 ± 0.17	1.4 × 10^−5^	0.374
	H_2_O_2_ (0.5 mM)	5.8 ± 0.15	4.6 ± 0.12	6.0 ± 0.15	4.1 × 10^−4^	0.184

Mean ± SD (*n* = 3) values (detected after 5 days of cultivation) are presented. ^b^—The colony diameter of the reference strain (Af293) was compared with that of the *ΔzipV* gene deletion mutant (VkzipV1) or the complemented strain (*C-zipV*; VkzipV2) with a two-sided, two-sample Student’s *t*-test. Values of *p* < 0.05 were considered to indicate a significant difference. Colony diameter data (cm) with the VkzipV22 complemented strain were as follows: 5.8 ± 0.1 (untreated), 4.2 ± 0.06 (MSB), 3.0 ± 0.1 (tBOOH) and 4.8 ± 0.2 (H_2_O_2_). Since these data did not differ significantly (*p* < 0.05) either between the two complemented strains or in comparison with the reference strain on 10 g/L glucose, this strain was not tested in further experiments. ^c^—In the bottom half of Table 1, g/L glucose was used (instead of 10 g/L) as a carbon/energy source during the preparation of the media.

## Data Availability

The transcriptome datasets are available in the Gene Expression Omnibus database (GEO; http://www.ncbi.nlm.nih.gov/geo/) under accession number GSE311793.

## Supplementary Materials

The following supporting information can be downloaded at: https://www.mdpi.com/article/10.3390/jof12050337/s1, Figure S1: PCR verification of *zipV* and *zipZ* gene deletions and *zipV* complementation using genomic DNA as template; Figure S2: Conidia of the *A. fumigatus* Af293, VKzipV1 and VKzipV2 strains. Table S1: Primer pairs used in this study; Table S2: RT–qPCR verification of *zipV* and *zipZ* expression in deletion, complementation, and wild-type strains; Table S3: Stress-tolerance attributes of the *A. fumigatus* ∆*zipV* strain; Table S4: Stress-tolerance attributes of the *A. fumigatus* ∆*zipZ* strain; Table S5: Evaluation of transcriptome data.

## Abbreviations

The following abbreviations are used in this manuscript:

bZIP	Basic Leucine Zipper
CP	Crossing Point
DEG	Differentially Expressed Gene
DFP	Deferiprone
MSB	Menadione Sodium Bisulfite
PBS	Phosphate-Buffered Saline
PCA	Principal Component Analysis
RIA	Reductive Iron Assimilation
ROS	Reactive Oxygen Species
RPKM	Reads Per Kilobase per Million
tBOOH	*tert*-Butyl Hydroperoxide
