# Supplementary material for: ZipV Is Required for Oxidative Stress Resistance and Pathogenicity in Aspergillus fumigatus
Source: J Fungi (Basel). 2026 May 5;12(5):337. doi: 10.3390/jof12050337 (PMC13208465; doi:10.3390/jof12050337)
Supplement: Supplementary file 1 [file jof-12-00337-s001.zip › Figure S1.pdf]

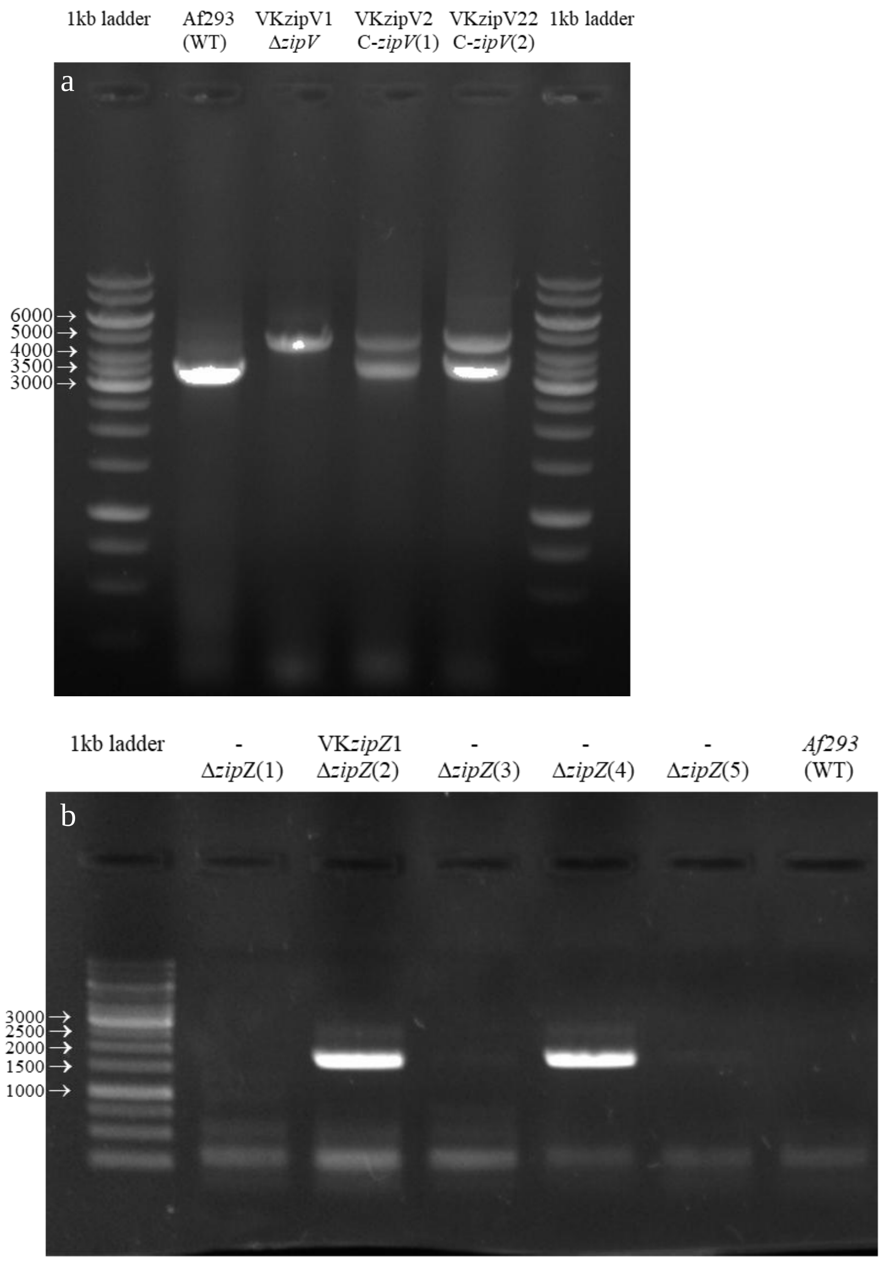

Figure S1 PCR verification of *zipV* and *zipZ* gene deletions and *zipV* complementation using genomic DNA as template.

Panel a: The primer pair zipV_HiFi_UP_F and zipV_Comp_Ble_R was used. The expected PCR product sizes are 3709 bp for the wild-type strain, 4917 bp for the Δ*zipV* mutant, and both fragments for the complemented strains.

Panel b: The primer pair 14350_UPchk_F and HygB_chk_R was used. These primers amplify a 1788 bp fragment spanning the genomic region upstream of the *zipZ* upstream homology arm and extending into the hygromycin resistance cassette, which is expected to be present only in the *zipZ* deletion strain.
