# Supplementary material for: ZipV Is Required for Oxidative Stress Resistance and Pathogenicity in Aspergillus fumigatus
Source: J Fungi (Basel). 2026 May 5;12(5):337. doi: 10.3390/jof12050337 (PMC13208465; doi:10.3390/jof12050337)
Supplement: Supplementary file 1 [file jof-12-00337-s001.zip › Figure S2 new.pptx]

## Slide 1
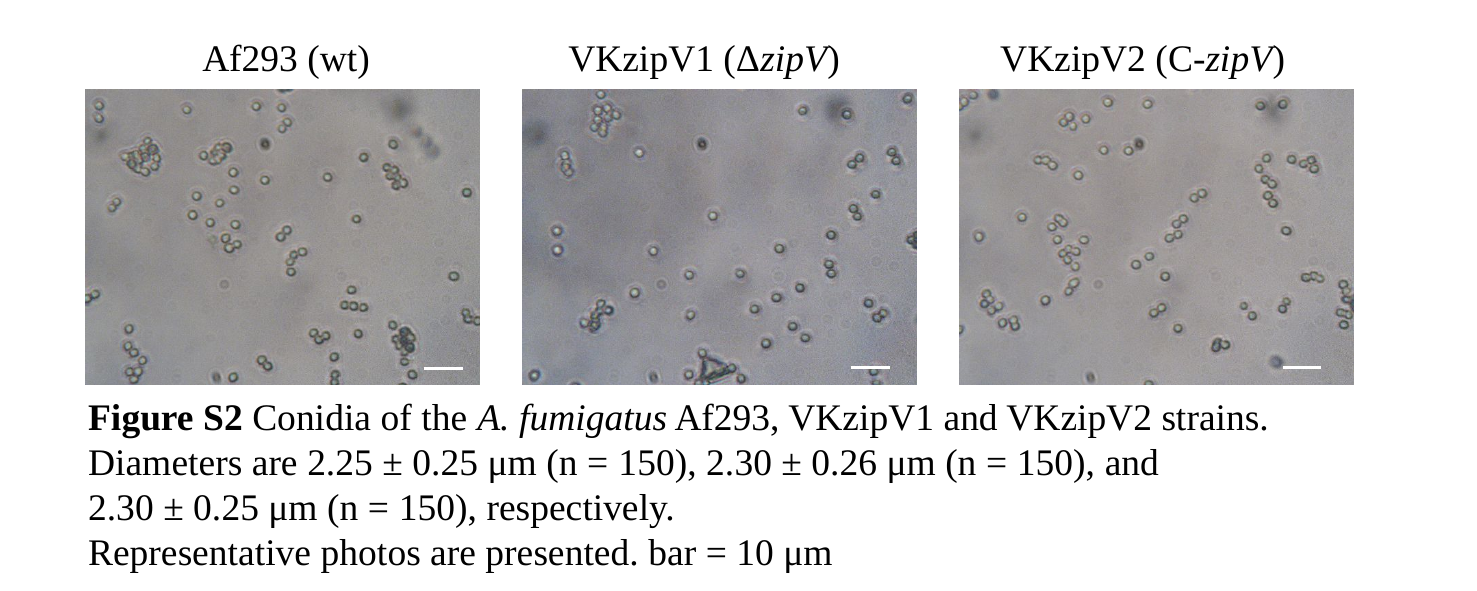

Af293 (wt) VKzipV1 (ΔzipV) VKzipV2 (C-zipV)
Figure S2 Conidia of the A. fumigatus Af293, VKzipV1 and VKzipV2 strains.
Diameters are 2.25 ± 0.25 μm (n = 150), 2.30 ± 0.26 μm (n = 150), and
2.30 ± 0.25 μm (n = 150), respectively.
Representative photos are presented. bar = 10 μm
