# Supplementary material for: ZipV Is Required for Oxidative Stress Resistance and Pathogenicity in Aspergillus fumigatus
Source: J Fungi (Basel). 2026 May 5;12(5):337. doi: 10.3390/jof12050337 (PMC13208465; doi:10.3390/jof12050337)
Supplement: Supplementary file 1 [file jof-12-00337-s001.zip › Table S1.pdf]

Table S1

Primer pairs used for gene deletions, complementation, PCR verification, and RT-qPCR assays

	Forward (5'-3')	Reverse (5'-3')
**For gene deletion cassettes:**		
php gene (HiFi_Hyg_F; HiFi_HygB_R)	CGAGCTCCCAAATCTGTCCAGATC	AGCTTGCATGCCTGCAGGTC
upstream region of *zipV* (*zipV*_HiFi_UP_F; *zipV*_HiFi_UP_R)	ATTCTGGTGGAACTGGATGGTGATGTCCTTGCCATTTCATCTAATG	CATGATCTGGACAGATTTGGGAGCTCGGATGACAACTTGAAGTTTATG
downstream region of *zipV* (*zipV*_HiFi_DW_F; *zipV*_HiFi_DW_R)	TCCACTCGACCTGCAGGCATGCAAGCTCACATTTTTACGTTATTCAG	GCCACGCACGGAAAACTTATGACCGTTTTGTGATTATTTGTGGAATG
gene deletion cassette (*zipV*_UP_F; *zipV*_DW_R)	CGGCGTCAGTTTGTCCAATTAC	CCGGGGCATTGTGATTATTTGTG
upstream region of *zipZ* (*zipZ*_HiFi_UP_F; *zipZ*_HiFi_UP_R)	ATTCTGGTGGAACTGGATGGTGATGTCATCGATGAATTCACTGACCTCGA	CATGATCTGGACAGATTTGGGAGCTCGCGTAGATATGTTGGAATCGTCCTC
downstream region of *zipZ* (*zipZ*_HiFi_DW_F; *zipZ*_HiFi_DW_R)	TCCACTCGACCTGCAGGCATGCAAGCTGCATGGATGATGGAACTGCCT	GCCACGCACGGAAAACTTATGACCGTTGCGCCCCATTGTTACCATAGTC
gene deletion cassette (*zipZ*_UP_F; *zipZ*_DW_R)	ATCTCCTCGGCCATCTCCAG	TAAAAGTCCAAGGCGCCATCG
**For complementation cassette:**		
ble gene (BleR_F, BleR_001_R)	CTCGTCCACCCCAACGCGTTT	GCCACGCACGGAAAACTTATGACCGTTAACAGTGCAATTATCTTTGCGAACC
zipV gene (*zipV*_HiFi_UP_F, *zipV*_Comp_Ble_R)	ATTCTGGTGGAACTGGATGGTGATGTCAGCTACGCACAATCCATCGGG	TGATAAACGCGTTGGGGTGGACGAGTCCGTTCCGATGGTATACCC
complementation cassette (*zipV*_HiFi_UP_F, BleR_001_R)	ATTCTGGTGGAACTGGATGGTGATGTCAGCTACGCACAATCCATCGGG	GCCACGCACGGAAAACTTATGACCGTTAACAGTGCAATTATCTTTGCGAACC
**For PCR verification:**		
zipV gene (*zipV*_HiFi_UP_F, *zipV*_Comp_Ble_R)	ATTCTGGTGGAACTGGATGGTGATGTCCTTGCCATTTCATCTAATG	TGATAAACGCGTTGGGGTGGACGAGTCCGTTCCGATGGTATACCC
zipZ gene (14350_UPchk_F; HygB_chk_R)	TGAACTTCACACCTGCATCG	AGAGTCACCGGTCACTGTAC

	Forward (5’-3’)	Reverse (5’-3’)
**For RT-qPCR assays:**		
catA gene (Afu6g03890)	CTTGTGCCCCTTCGTGTG	GCCAGTAGTTGACCGTGCC
rodA gene (Afu5g09580)	TCTCTTCAACCAGTGCTCCA	CTGCTTGCACTTCTGGTTGA
zipV gene (Afu3g03230)	GGTCATCGAGACAATGACGG	ATACTTGGCGGTCACCTCTT
zipZ gene (Afu2g14350)	CGTGTCGTCCCCTAGCGAGGAA	GTCCCCAAACCTTGGCTTCCCG
aoxA gene (Afu2g05060)	AGGGACAACGGATGGATTGA	CTCAGCAAGCTTGAGGAAGG
tef1 gene (Afu1g06390)	AAGTCCGAGCGTGAGCGTG	GGTGAAAGCGAGCAGAGCG
