# Supplementary material for: ZipV Is Required for Oxidative Stress Resistance and Pathogenicity in Aspergillus fumigatus
Source: J Fungi (Basel). 2026 May 5;12(5):337. doi: 10.3390/jof12050337 (PMC13208465; doi:10.3390/jof12050337)
Supplement: Supplementary file 1 [file jof-12-00337-s001.zip › Table S2.pdf]

Table S2 RT–qPCR verification of *zipV* and *zipZ* expression in deletion, complementation, and wild-type strains.

	Transcriptional activity (CP)[a]				
	Af293 (wt)	VKzipV1 (Δ*zipV*)	VKzipV2 (c-*zipV*)	VKzipV22 (c-*zipV*)	VKzipZ1 (Δ*zipZ*)
zipV (Afu3g03230)	26.2 ± 0.5	39.3 ± 1.0	27.0 ± 1.0	27.8 ± 0.8	n.d.
zipZ (Afu2g14350)	24.5 ± 0.6	n.d.	n.d.	n.d.	37.8 ± 1.0

[a] – Barratt's minimal liquid medium supplemented with 5 g/L yeast extract was inoculated with 5×10^7 conidia, and incubated at 37°C and 220 rpm (approximately 3.7 Hz) for 17 h, to obtain cultures in the exponential growth phase. Total RNA was isolated from lyophilized mycelia using the acid guanidinium thiocyanate-phenol-chloroform extraction method. Mean ± SD of four biological replicates are presented. CP stands for crossing point of the RT-qPCR reaction.

n.d. – not determined
