# Supplementary material for: ZipV Is Required for Oxidative Stress Resistance and Pathogenicity in Aspergillus fumigatus
Source: J Fungi (Basel). 2026 May 5;12(5):337. doi: 10.3390/jof12050337 (PMC13208465; doi:10.3390/jof12050337)
Supplement: Supplementary file 1 [file jof-12-00337-s001.zip › Table S3.pdf]

Table S3 Characterization of stress tolerance attributes of *A. fumigatus ΔzipV* strain

	Colony diameter (cm)[a]		*p*-value[b]
	Af293	*ΔzipV*	
Untreated	6.0 ± 0.10	5.9 ± 0.10	0.140
Sorbitol (1 M)	3.7 ± 0.23	3.4 ± 0.08	0.095
NaCl (0.5 M)	5.0 ± 0.53	4.7 ± 0.67	0.617
Congo red (15 mM)	3.3 ± 0.00	3.3 ± 0.08	0.725
DFP (1 mM)[c]	3.4 ± 0.23	3.3 ± 0.05	0.431
$ZnSO_4$ (8 mM)	4.2 ± 0.10	4.0 ± 0.12	0.057
$FeCl_3$ (6.5 mM)	2.6 ± 0.32	3.0 ± 0.55	0.338
$CuCl_2$ (0.5 mM)	1.6 ± 0.03	1.6 ± 0.09	0.768
$CdCl_2$ (2 mM)	3.5 ± 0.06	3.4 ± 0.10	0.116

[a] – Mean ± SD (n = 3) values (detected after 5 days cultivation) are presented.

[b] – The colony diameter of the reference strain (Af293) was compared with that of the gene deletion mutant (*ΔzipZ*) with two-sided, two-sample Student's t-test. No significant ($p < 0.05$) differences between strains were found under any of the culture conditions.

[c] – During preparation of these media iron was omitted from the trace element solution.
