# Supplementary material for: ZipV Is Required for Oxidative Stress Resistance and Pathogenicity in Aspergillus fumigatus
Source: J Fungi (Basel). 2026 May 5;12(5):337. doi: 10.3390/jof12050337 (PMC13208465; doi:10.3390/jof12050337)
Supplement: Supplementary file 1 [file jof-12-00337-s001.zip › Table S4.pdf]

Table S4 Characterization of stress tolerance attributes of *A. fumigatus ΔzipZ* strain

	Colony diameter (cm)[a]		*p*-value[b]
	Af293	*ΔzipZ*	
Untreated	6.0 ± 0.16	6.0 ± 0.03	0.240
Sorbitol (1 M)	4.0 ± 0.17	3.9 ± 0.15	0.374
NaCl (0.5 M)	4.7 ± 0.10	4.4 ± 0.15	0.074
Congo red (15 mM)	3.7 ± 0.18	3.5 ± 0.15	0.289
DFP (1 mM)[c]	3.0 ± 0.25	3.0 ± 0.08	0.917
$ZnSO_4$ (8 mM)	4.2 ± 0.18	4.3 ± 0.22	0.633
$FeCl_3$ (6.5 mM)	3.1 ± 0.15	2.9 ± 0.10	0.091
$CuCl_2$ (0.5 mM)	1.3 ± 0.10	1.3 ± 0.03	0.468
$CdCl_2$ (2 mM)	3.7 ± 0.10	4.2 ± 0.33	0.065
MSB (6 mM)	4.2 ± 0.21	3.9 ± 0.15	0.169
H_2O_2 (0.5 mM)	4.4 ± 0.25	4.2 ± 0.00	0.276
tBOOH (0.8 mM)	3.4 ± 0.20	3.2 ± 0.95	0.679

[a] – Mean ± SD (n = 3) values (detected after 5 days cultivation) are presented.
[b] – The colony diameter of the reference strain (Af293) was compared with that of the gene deletion mutant (*ΔzipZ*) with two-sided, two-sample Student's t-test. No significant ($p < 0.05$) differences between strains were found under any of the culture conditions.
[c] – During preparation of these media iron was omitted from the trace element solution.
